# *Bacillus thuringiensis* Cyt2Aa2 toxin disrupts cell membranes by forming large protein aggregates

**DOI:** 10.1042/BSR20160090

**Published:** 2016-10-14

**Authors:** Sudarat Tharad, José L. Toca-Herrera, Boonhiang Promdonkoy, Chartchai Krittanai

**Affiliations:** * Institute of Molecular Biosciences, Mahidol University, Salaya Campus, Putthamonthon 4 Rd., Salaya, Nakhonpathom 73170, Thailand; † Department of Nanobiotechnology, Institute for Biophysics, University of Natural Resources and Life Sciences Vienna (BOKU), Muthgasse 11, Vienna 1190, Austria; ‡ National Center for Genetic Engineering and Biotechnology (BIOTEC), National Science and Technology Development Agency (NSTDA), 113 Phahonyothin Rd., Pathumthani 12120, Thailand

**Keywords:** *Bacillus thuringiensis*, complex formation, cytolytic activity, protein aggregation

## Abstract

*Bacillus thuringiensis* (*Bt*) Cyt2Aa2 showed toxicity against Dipteran insect larvae and *in vitro* lysis activity on several cells. It has potential applications in the biological control of insect larvae. Although pore-forming and/or detergent-like mechanisms were proposed, the mechanism underlying cytolytic activity remains unclear. Analysis of the haemolytic activity of Cyt2Aa2 with osmotic stabilizers revealed partial toxin inhibition, suggesting a distinctive mechanism from the putative pore formation model. Membrane permeability was studied using fluorescent dye entrapped in large unilamellar vesicles (LUVs) at various protein/lipid molar ratios. Binding of Cyt2Aa2 monomer to the lipid membrane did not disturb membrane integrity until the critical protein/lipid molar ratio was reached, when Cyt2Aa2 complexes and cytolytic activity were detected. The complexes are large aggregates that appeared as a ladder when separated by agarose gel electrophoresis. Interaction of Cyt2Aa2 with *Aedes albopictus* cells was investigated by confocal microscopy and total internal reflection fluorescent microscopy (TIRF). The results showed that Cyt2Aa2 binds on the cell membrane at an early stage without cell membrane disruption. Protein aggregation on the cell membrane was detected later which coincided with cell swelling. Cyt2Aa2 aggregations on supported lipid bilayers (SLBs) were visualized by AFM. The AFM topographic images revealed Cyt2Aa2 aggregates on the lipid bilayer at low protein concentration and subsequently disrupts the lipid bilayer by forming a lesion as the protein concentration increased. These results supported the mechanism whereby Cyt2Aa2 binds and aggregates on the lipid membrane leading to the formation of non-specific hole and disruption of the cell membrane.

## Abstract (precis)

We show that the lipid membrane disruption by *Bacillus thuringiensis* (*Bt*) Cyt2Aa2 is different from the general pore-forming model. Cyt2Aa2 forms protein aggregates that disrupt the lipid membrane integrity.

## INTRODUCTION

*Bacillus thuringiensis* (*Bt*) is an aerobic gram-positive bacterium, which produces insecticidal proteins during the sporulation phase [1]. These insecticidal proteins are parasporal crystals consisting of two delta-endotoxin families, crystal (Cry) and cytolytic (Cyt) toxins [2]. Although the toxins exhibit larvicidal activity, Cry and Cyt proteins share no homology in their amino acid sequences, and adopt an entirely different structure [3,4]. Cyt toxins are classified into three classes, Cyt1, Cyt2 and Cyt3 based on their amino acid identity [5]. Cyt2Aa2 is produced from *Bt* subsp. *darmstadiensis* as a 259-amino acid sequence protoxin [6]. The 3D structure of inactivated Cyt2Aa shows a monomeric structure with high similarity to protease-activated Cyt2Ba [7], with their secondary and tertiary structures being very similar. These crystallographic structures suggest a respective conformational change of the active toxin when binding to lipid bilayers. Activation of Cyt toxin generally takes place through proteolytic processing to remove amino acids from the N- and C-termini of the protein [8]. The toxin exhibits cytolytic activity *in vitro* towards *Escherichia coli* and *Staphylococcus aureus* cells [9] and also towards a variety of insect and mammalian cells, including erythrocytes, lymphocytes and fibroblasts [10,11]. However, it shows specific *in vivo* toxicity against Dipteran insect larvae, such as mosquitoes and black flies [12,13].

Cyt toxin can bind with unsaturated phospholipids such as phosphatidylcholine, phosphatidylethanolamine and sphingomyelin on cell membranes [11,14,15]. Planar lipid bilayer experiments using Cyt1A suggested the formation of ionic channels or pores in the membrane [16]. In addition, acrylodan-labelled Cyt2Aa1 toxins showed the presence of labelled-cysteine residues, either buried in the hydrophobic core or inserted into the membrane [17]. Alternatively, liposome binding and fluorescent dye releasing assays suggested a large number of Cyt1A toxins adsorbed on to the lipid membrane. Membrane permeability was enhanced through the perturbation of the lipid membrane in a detergent-like action, leading to the release of marker molecules of different sizes [18,19]. These ambiguous results led to two possible models for the cytolytic mechanism. The pore-forming model proposed that each monomer came together to form an oligomeric pore in the lipid bilayer membranes [3,17,20–22]. The detergent-like model suggested aggregation of toxin monomers on the surface of the lipid bilayers until they reached a critical concentration at which point the lipid membrane was then disrupted by a detergent-like activity [23,24]. However, a definitive mechanism for the cytolytic activity of the Cyt toxin remains unclear.

The present study aimed to investigate the lipid membrane disruption by Cyt2Aa2 based on an analysis of haemolytic activity in the presence of an osmotic stabilizer. Cyt2Aa2 complex formation and lipid membrane perturbation were investigated on the large unilamellar vesicles (LUVs) and *Aedes albopictus* cells using fluorescent dye detection. An inactive N145A mutant toxin was used as a control in this work. An alanine substitution was introduced into the position Asn-145 in the loop between αD-β4. Structural characterization revealed similar folding and biochemical properties to that of wild type. Membrane interaction assays previously show that N145A mutant did not bind and form complexes on liposomes, sheep erythrocytes and brush border membrane fractions (BBMF) from *Aedes aegypti* larvae [32]. Moreover, topographic images of membrane disruption were analysed by AFM. Our results suggested that the lipid membrane disruption by Cyt2Aa2 occurs after binding, with the formation of protein aggregations and a subsequence disruption rather than through pore formation followed by cell swelling and lysis.

## MATERIALS AND METHODS

### Expression and purification of Cyt2Aa2 toxin

Cyt2Aa2 toxin was expressed from *E. coli* cell culture and the expressed inclusion protein was harvested as previously described by Thammachat et al. [25] and Promdonkoy and Ellar [17]. The isolated inclusion was solubilized in 50 mM Na_2_CO_3_ pH 10.0 at 37°C for 1 h and soluble toxin was obtained after centrifugation to remove insoluble material at 12,000 ***g*** for 10 min.

For proteolytic activation, the soluble toxin was incubated with 2% (w/w) chymotrypsin (Sigma) at 37°C for 2 h. Both protoxin and activated Cyt2Aa2 were purified by anion exchange chromatography using a 1 ml HiTrap Q XL column (GE Healthcare). Purified proteins were obtained by elution with a linear gradient of 0−0.5 M NaCl in 50 mM Tris-base pH 10.0 at flow rate of 0.5 ml/min. Salt was removed by dialysis in 50 mM Na_2_CO_3_ pH 10.0 using cellulose membrane tubing, 10 kDa MWCO (Spectra/Por). Protein concentrations were determined by UV absorption at 280 nm (NanoDrop 1000) with an molar absorption coefficient for Cyt2Aa2, *ε*_protoxin_=0.852 (mg/ml)^−1^·cm^−1^ and *ε*_activated toxin_=0.891 (mg/ml)^−1^·cm^−1^. The concentrations of purified protoxin and activated toxin were approximately 3 mg/ml.

### Preparation of fluorescent-labelled protoxin

Purified Cyt2Aa2 protoxin at 2 mg/ml in Na_2_CO_3_ buffer was incubated with 10 mg/ml of Texas Red-X succinimidyl ester dissolved in DMSO at weight ratio of 2:1. The conjugation reaction was performed at 25°C for 1 h with continuous shaking at 600 rpm. The free dye was removed by PD-10 column (GE Healthcare). The labelled protein was protected from light during the conjugating reaction and purification steps. Protein concentration and degree of labelling were calculated according to product instructions (eqns 1 and 2). Successful labelling was determined by SDS/PAGE and size-exclusion chromatography using a Superdex200 10/300 GL column (GE Healthcare). The final protein concentration of labelled protoxin is 1 mg/ml.

The protein concentration and degree of labelling of labelled Cyt2Aa2 were calculated by the following equations:

1 $$\begin{array}{ccc} & & \mathrm{Protein}\;\mathrm{concentration}\;\left(\mathrm{mg}/\mathrm{ml}\right)\;\\ & & \;=\;\frac{\left[A280-\left(A595\cdot \mathrm{CF}\right)\right]\cdot \mathrm{dilution}\;\mathrm{factor}}{ɛ} \end{array}$$

CF=correction factor of Texas red; 0.18

*ε*=molar absorption coefficient of protoxin; 0.852 (mg/ml)^−1^·cm^−1^

2 $$\begin{array}{ccc} & & \mathrm{Degree}\;\mathrm{of}\;\mathrm{labelling}\;\left(\mathrm{mole}\;\mathrm{dye}\;\mathrm{per}\;\mathrm{mole}\;\mathrm{protein}\right)\;\\ & & \;=\;\frac{A595\cdot \mathrm{dilution}\;\mathrm{factor}}{{}_{{}^{ɛ}\mathrm{Texas}\;\mathrm{red}}\cdot \mathrm{Protein}\;\mathrm{conc}.} \end{array}$$

*ε*_Texas red_=molar absorption coefficient of Texas red at 595 nm, 80000 M^−1^·cm^−1^

Protein conc.=protein concentration of Cyt2Aa2 in molar.

### Preparation of multilamellar liposomes

Multilamellar liposomes were prepared according to the method described by Thomas and Ellar [11] with some modifications. Egg yolk phosphatidylcholine, cholesterol and stearylamine (Sigma) dissolved in chloroform were mixed at a molar ratio of 4:3:1 to a total lipid of 10 mg and dried under a gaseous nitrogen (N_2_) stream. The lipid film was rehydrated to a final concentration of 10 mg/ml by 50 mM Na_2_CO_3,_ pH 10.0 and then sonicated for 10 min. Liposome aliquots of 1 mg were kept at −80°C until required for use.

### Preparation of calcein-entrapped large unilamellar vesicle

A 4:3:1 mixture of phosphatidylcholine, cholesterol and stearylamine (Sigma) in chloroform was prepared at a total lipid content of 2 mg and dried under N_2_. The lipid film was rehydrated with 60 mM calcein (Invitrogen) dissolved in 50 mM Na_2_CO_3_, pH 10.0 at 40°C for 1 h. The prepared vesicles were repeatedly extruded through a 100 nm polycarbonate membrane using a mini-extruder (Avanti), and free calcein was removed by a 5 ml desalting column (GE Healthcare). The concentration of LUVs was determined by a phosphorous assay [26].

### SDS/PAGE and immunodetection

Protein samples were mixed with protein loading buffer and separated by SDS/PAGE using 12.5% polyacrylamide gel and then transferred on to nitrocellulose membrane by wet-blotting technique using transfer buffer (193.0 mM glycine, 24.8 mM Tris-base, 1.4 mM SDS and 20% (v/v) methanol). Non-specific protein binding was prevented by 5% skim milk in PBS pH 7.4 (137.0 mM NaCl, 2.7 mM KCl, 10.0 mM Na_2_HPO_4_ and 2.0 mM KH_2_PO_4_) at 4°C for at least 2 h. The membrane was incubated with anti-Cyt2Aa2 IgG (1:8000) at room temperature for 1.5 h, and washed with PBS pH 7.4 containing 0.1% Tween 20 for 5 min, three times. The membrane was then incubated with secondary antibody, goat anti-rabbit IgG, conjugated horseradish peroxidase (1:10000) (Kirkegaard & Perry Laboratories) at room temperature for 1.5 h and washed with PBS pH 7.4 containing 0.1% Tween 20 for 5 min, three times. Blotted proteins were detected by adding ECL substrate (Thermo scientific pierce) for 5 min and exposed to film.

### Analysis of permeable activity and protein complex formation

Calcein-entrapped LUVs of 5 μg (11.2 nmol) were incubated with activated toxin of various amounts from 80 μg (3.2 nmol) to 0.63 μg (0.034 nmol) at a final volume of 500 μl, 25°C for 1 h. The permeable activity of Cyt2Aa2 was determined by measuring the fluorescent emission of calcein at 520 nm with excitation at 485 nm. The activity was expressed as a percentage of the total calcein released after adding 0.074% Triton X-100, using the equation: % Calcein release=(*F*_p_ − *F*_0_ /*F*_T_ − *F*_0_) x100, where *F*_p_ is the intensity by toxin treatment, *F*_T_ is the intensity by Triton X-100 treatment and *F*_0_ is the baseline fluorescence of the LUVs. The protein complexes forming on LUVs were separated by centrifugation at 15000 ***g***, 4°C for 30 min. The pellet was incubated with protein loading buffer at room temperature for 10 min, analysed by SDS/PAGE and visualized by silver staining [27].

### Haemolytic activity assay

Erythrocytes from sheep blood (National Laboratory Animal Center, Mahidol University) were isolated by centrifugation at 3000 ***g*** for 5 min. The purified erythrocytes were washed twice with PBS pH 7.4 and re-suspended in PBS, or PBS containing 9% (w/v) PEG 400, or PBS containing 20% (w/v) PEG4000. The percentage of PEG in solution was adjusted to obtain equal osmotic pressure [28]. Activated Cyt2Aa2 was prepared in each solution used for erythrocyte suspension. The 2% (v/v) erythrocytes of 200 μl were incubated with 200 μl activated Cyt2Aa2 of various concentrations at 25°C for 2 h. Haemolytic activity was assessed from the haemoglobin released. The treated erythrocytes were centrifuged at 10000 ***g*** for 1 min at 4°C, and then 200 μl of supernatant containing haemoglobin was transferred to a flat bottom 96-well plate. The blank was PBS or PBS containing PEG. A 100% haemolysis was obtained from the supernatant of 0.1% (v/v) Triton X-100 treatment. Haemoglobin absorption was monitored at 540 nm. The haemolytic activity was calculated as a percentage of the haemolysis described by the equation; %Haemolysis=(*H*_p_ − *H*_0_/*H*_T_ − *H*_0_) × 100, where *H*_p_ is haemolysis caused by protein, *H*_T_ is haemolysis caused by Triton X-100 and *H*_0_ is the absorbance from non-treated erythrocytes. Statistical analysis was performed by using the concentration of protein stock prepared in triplicate. S.D. and S.E.M., where 
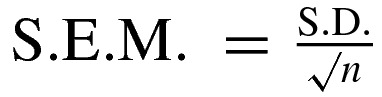
and *n* is number of sample, were then calculated. The protein concentration at 50% haemolysis of each treatment was analysed for statistical difference by one-way ANOVA with 95% confidence.

### Study of protein complex formation at specific molar rati*o*

Large multilamellar liposomes of 100 μg (224 nmol) were incubated with activated Cyt2Aa2 at various protein/lipid molar ratios at a total volume of 260 μl, 25°C for 1 h. The membrane-bound protein was separated by centrifugation at 15000 ***g***, 4°C for 30 min and resuspended in 200 μl of PBS pH 7.4. Cross-linking reaction was performed in 0.1% (v/v) glutaraldehyde (Sigma) at 25°C for 10 min and stopped by 20 μl of 0.5 M Tris/HCl pH 7.5. The protein complex was collected by centrifugation at 15000 ***g***, 4°C for 30 min and mixed with protein loading buffer. After heating at 95°C for 5 min the sample was analysed by vertical SDS–agarose gel electrophoresis. The method was similar to conventional SDS/PAGE, except that polyacrylamide was substituted with 0.8% (w/v) agarose in 0.375 M Tris/HCl, pH 8.8 (Vivantis). The protein complex was separated by electrophoresis and detected by immunodetection.

### Mosquito cell culture

*A. albopictus* cells (A.T.C.C.: CRL-1660) were maintained at 28°C in Leibovitz's L-15 medium (HyClone), supplemented with 10% heat inactivated FBS (Gibco) and 100 unit/ml of penicillin/streptomycin (PAA Laboratories GmbH). The cell was maintained in 25 cm^2^ tissue culture flasks (Corning) and subcultured by detaching cells with 0.25% trypsin/EDTA solution. For the treatment experiments, 1 ml of 1:5 cell dilution was seeded on to 15 mm round cover slips (Menzel-Glaszer) in 12-well plates (Nunc) or 8-well culture slide cover glass (LabTek II). The cells were allowed to adhere on to cover slips at 28°C for 48 h with approximated cell density of 80%.

### Analysis of cell membrane binding

In a time-lapse experiment, *A. albopictus* cells cultured on cover glass were incubated with active labelled Cyt2Aa2 at a protein concentration of 10 μg/ml. The nucleus was stained by 5 μg/ml Hoechst in Leibovitz's L-15 medium at 25°C, 30 min before the experiment. The binding of labelled toxin on the cell membrane was monitored using an Olympus FV10i confocal microscope.

### Total internal reflection fluorescent microscopy

*A. albopictus* cells cultured on slide cover glass were treated with 10 μg/ml of active labelled Cyt2Aa2 in Leibovitz's L-15 medium at room temperature. After treatment the cells were rapidly fixed with 4% paraformaldehyde and mounted with 1,4-diazobizyclo(2,2,2) octane (DABCO) antifade. Cell membrane image was monitored by Olympus TIRFM using a 100×,1.49 NA objective lens. Fluorophore molecules were excited by 640 nm laser (red signal). The images were processed by ImageJ programme.

### AFM

Supported lipid bilayers (SLBs) were formed by a liposome fusion method. Briefly, 50 nm LUVs (13:1 POPC:cholesterol in molar ratio) (Sigma) were prepared by extrusion. 0.1 mg/ml of LUVs were incubated with UV/Ozone-treated silicon wafer (0.49 cm^2^) (IMEC, Leuven) at room temperature for 30 min. The intact LUVs were flushed from the lipid bilayer surface. Subsequently, 200 μl of the desired protein concentrations of activated Cyt2Aa2 were incubated with SLBs at room temperature for 1 h. The excess protein was removed and then the protein–lipid complexes were visualized by AFM technique with a J-scanner controlled by NanoScope V multimode software (Bruker). Bio-Lever mini silicon nitride probe BL-AC40TS (Olympus) with resonance frequency of 25 kHz (in liquid) and spring constant of 0.09 N/m is used in tapping mode. Prior to its use, the probes were cleaned with UV/Ozone for 20 min. Once mounted, the system was maintained until stabilization of the deflection signal. The AFM images were obtained in tapping mode with a scanning rate of 0.5–1.0 Hz. All the images were processed by the Nanoscope programme.

## RESULTS AND DISCUSSION

### Haemolytic activity of Cyt2Aa2 in colloidal osmotic stabilizer solution

**Figure 1:**
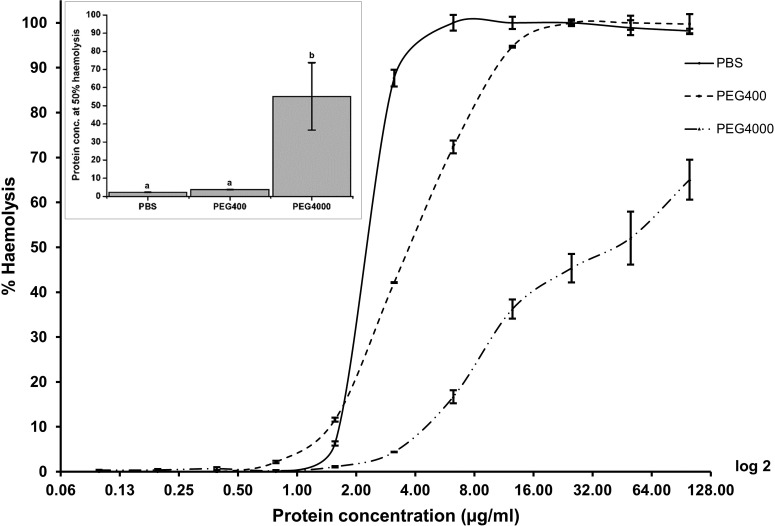
Haemolytic activity of Cyt2Aa2 in osmotic stabilizing solution Activated Cyt2Aa2 was incubated with sheep erythrocytes at 25°C for 2 h. The haemoglobin release was assessed by measuring absorbance at 540 nm. Each value represents mean ± S.E.M. (*n*=3). The protein concentrations inducing 50% haemolysis are shown in the bar graph showing mean ± S.E.M. (inset figure). The difference between the treatments was analysed by one-way ANOVA with 95% confidence. The same letter above the bar graph indicates no significant difference in statistical analysis.

When sheep erythrocytes were incubated with various concentrations of activated Cyt2Aa2 toxin in an isotonic PBS solution, haemolytic activity was observed as a sigmoidal curve (with logarithmic scale). A sharp increase in haemolysis from 6 to 100% was found for toxin concentrations ranging from 1.56 to 6.25 μg/ml (Figure 1). According to the general pore-forming model such as in Staphylococcal α-toxin, a number of well-defined pores on the lipid membrane were hypothesized [29]. These membrane pores of specific pore size can lead to haemolytic activity by colloidal osmotic imbalance. In our experiment PEG was added to the PBS solution to act as an osmotic stabilizing agent. Their stabilizing effect helped prevent colloidal osmotic haemolysis. We found that as an osmotic stabilizing agent, PEG400 did not show an inhibition effect on the haemolytic activity of Cyt2Aa2; whereas, PEG4000 could partially inhibit haemolysis. As such, a concentration of the toxin required to cause 50% haemolysis was significantly higher for solution containing PEG4000 than that of isotonic and PEG400 solutions (Figure 1 inset). Although haemolysis of incubated erythrocytes still increased with exposure to higher concentrations of toxin. However, altered haemolytic activity found in the PEG4000 solutions may reflect different pore sizes or leakages of the cell membrane in the two concentration ranges. Accordingly, at higher protein concentrations (≥6.25 μg/ml) where the haemolysis could be initially observed in PEG4000 solutions the toxin might form larger leakages of the lipid membrane than at lower concentrations (1.56–3.12 μg/ml). Previous studies have demonstrated that 10% (v/v) PEG1000 could not completely prevent haemolytic activity of 10 μg/ml Cyt2Aa1, which showed a haemolytic activity approximately 20%. Subsequently, when PEG1000 was removed from the isotonic solution, then the haemolysis was increased to almost 100% [15]. However, lower haemolytic activity of Cyt2Aa2 found in PEG solutions could be the result of the effect of PEGs on toxin conformation. The haemolysis in PEG solution could be explained differently for PEG400 and PEG4000. Cyt2Aa2 may form pores or leakages on the membrane that are large enough for PEG400 molecule (*R*_h_ ≈ 0.002 nm) to move into the cell and fail to prevent osmotic imbalance. On the contrary, at lower toxin concentrations the size of membrane leakages might be too small for PEG4000 (*R*_h_ ≈ 1.8 nm) to pass whereas at higher toxin concentrations, Cyt2Aa2 might form large membrane leakages, allowing either haemoglobin diffuse from the erythrocytes or PEG4000 diffuse into the cells. Our results are in agreement with the previous report in which the 24 kDa-Cyt1A at 260 nM (6.24 μg/ml) could induce the release of dextran 10000 (*R*_h_ ≈ 1.86 nm) from LUVs [18]. As a conclusion, the different effect of the two PEG compounds suggest that PEG 400 can permeate and lose its osmolality stabilizing function following toxin addition whereas PEG 4000 cannot permeate, giving an indication of the scale of pore/detergent-like hole sizes. Since haemolytic activity of Cyt2Aa2 was not completely inhibited by osmotic stabilizing solution, it has been suggested that haemolytic activity of Cyt2Aa2 may not rely only on the colloidal osmotic pressure. This mechanism would be distinct from true pore-forming proteins.

### Protein complex formation and calcein release assay

**Figure 2:**
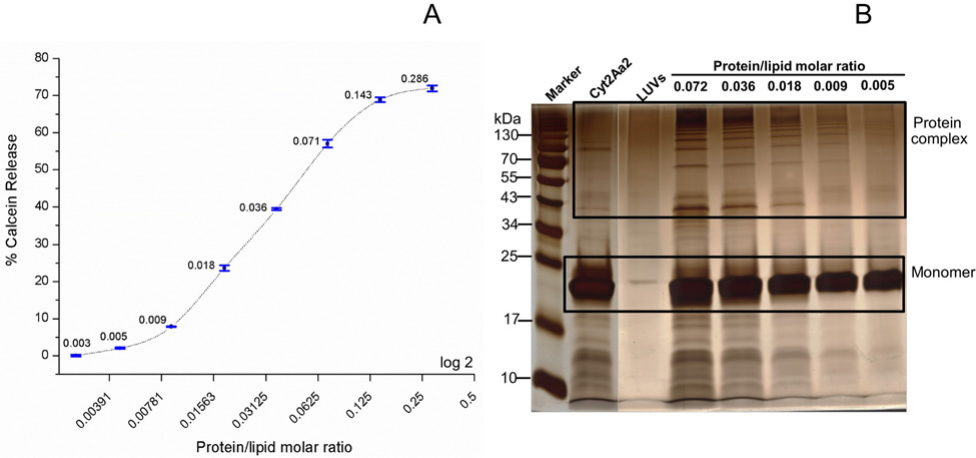
Relationship of calcein releasing activity (A) and protein complex formation (B) Activated Cyt2Aa2 was incubated with calcein-entrapped LUVs at protein/lipid molar ratios from 0.003 to 0.286 (**A**). Calcein release activity was measured at excitation wavelength 485 nm and emission wavelength 520 nm. Each data point represents mean ± S.E.M. (*n*=3). Protein complexes forming on LUVs at protein/lipid molar ratios from 0.005 to 0.072 were collected by centrifugation, analysed by 12.5% SDS/PAGE and visualized by silver staining (**B**).

Activated Cyt2Aa2 was incubated with calcein-entrapped LUVs at various protein/lipid molar ratios ranging from 0.005 to 0.286. The cytolytic activity of calcein release demonstrated a sigmoidal curve, with detectable activity starting from a protein/lipid molar ratio of 0.009 (Figure 2A). The activity increased and reached saturation at the protein/lipid molar ratio of 0.143. The protein complexes on the lipid membrane were then isolated from the reaction and analysed by SDS/PAGE. Although monomeric protein was found as a major band in various molar ratios, high molecular mass bands of membrane-bound protein complexes also appeared. These high molecular mass protein complexes were observed from protein/lipid molar ratios between 0.005 and 0.072 (Figure 2B). This range of molar ratio gave strong intensity of the calcein released from LUVs. Although the true pore-forming proteins/peptides achieved a 100% release of dye molecules at protein/lipid molar ratios much lower than 1:1000 [30], Cyt2Aa2 exerted maximal activity at the protein/lipid molar ratios of 1:200 or higher. Our data suggested that monomeric Cyt2Aa2 binds to the lipid membrane with inefficient membrane disruption. When the toxin accumulates to only a low critical number, membrane integrity is disrupted and the dye is released from the vesicles. This membrane disruption model may be similar to that proposed by Rodriguez-Almazan et al. [31] but in the case of monomeric Cyt1A the toxin bound at an early stage and then triggered membrane permeability by oligomerization and penetration; it is not possible to be conclusive in the case of Cyt2Aa2.

### Large complex formation at specific protein/lipid molar ratio

**Figure 3:**
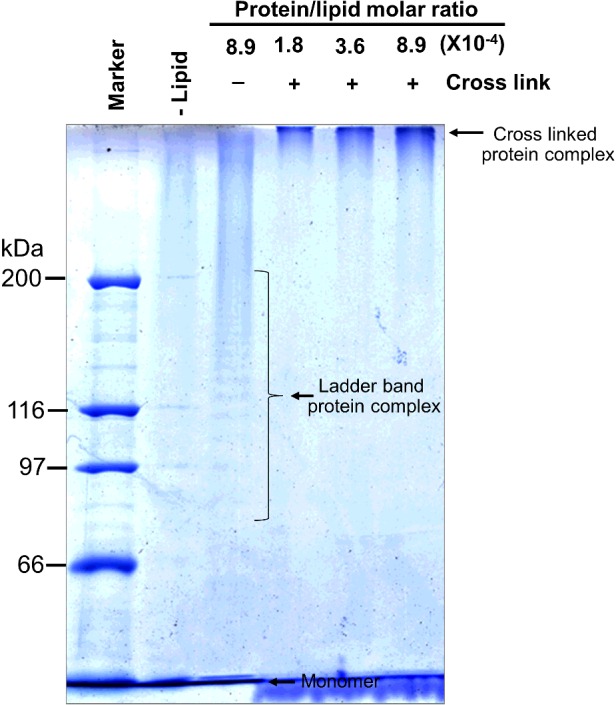
SDS/PAGE analysis of cross-linked protein complex of Cyt2Aa2 Activated Cyt2Aa2 was incubated with large multilamellar liposomes at various protein/lipid molar ratios at 25°C for 1 h. The membrane-bound protein was collected by centrifugation. The protein complex was cross-linked by 0.1% glutaraldehyde at 25°C for 10 min. The cross-linked protein complexes were separated by SDS/PAGE, 6% acrylamide gel (2.6% bisacrylamide) and detected by Coomassie blue staining.

**Figure 4:**
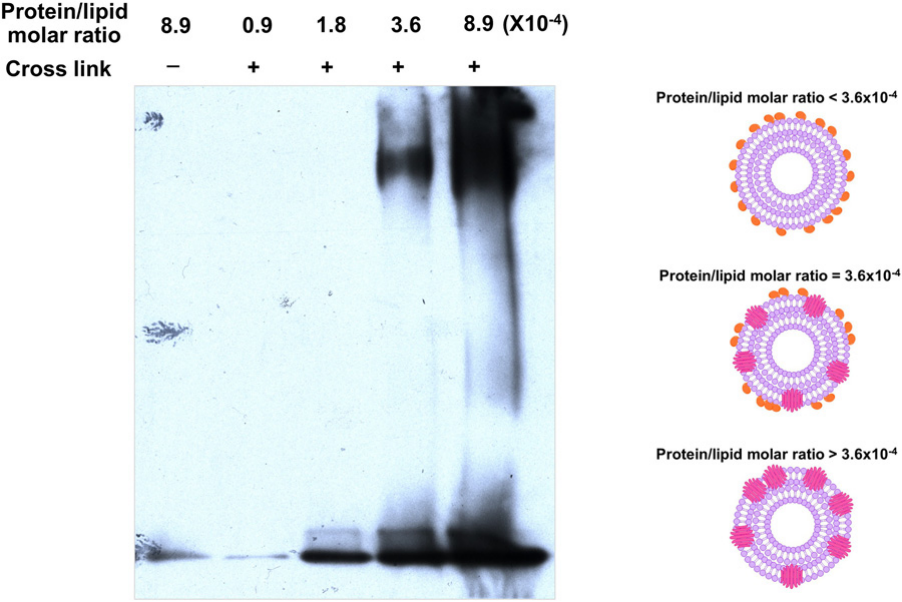
Protein complex formation at specific protein/lipid molar ratio Activated Cyt2Aa2 was incubated with large multilamellar liposomes at various protein/lipid molar ratios. The membrane-bound protein was collected by centrifugation. The protein complex was cross-linked by glutaraldehyde and separated by SDS–agarose gel (0.8% w/v). Cyt2Aa2 was detected by immunoblotting. The right panel shows a model for Cyt2Aa2 binding on lipid membrane at various protein/lipid molar ratios. At protein/lipid molar ratio lower than 3.6×10^−4^, Cyt2Aa2 binds to the lipid membrane as monomer which is not close enough for cross-linking by glutaraldehyde. When the protein/lipid molar ratio increases further to 3.6×10^−4^ or greater Cyt2Aa2 forms large protein complexes, where upon Cyt2Aa2 molecules are close enough for glutaraldehyde cross-linking.

The activated Cyt2Aa2 was incubated with large multilamellar liposomes at various molar ratios and subsequently separated by SDS/PAGE. The SDS-sensitive Cyt2Aa2 complex was protected from dissociation by covalent bond cross-linking with glutaraldehyde. The cross-linked Cyt2Aa2 complex stuck in the well when compared with the uncross-linked Cyt2Aa2 complex that showed a characteristic ladder-like band on SDS/PAGE (Figure 3). To separate the large Cyt2Aa2 complex, agarose gel electrophoresis was used to replace the polyacrylamide gel. The large protein complex was then observed as a streak at specific protein/lipid molar ratios between 3.6×10^−4^ and 8.9×10^−4^ (Figure 4). A large Cyt2Aa2 complex was detected at a protein/lipid molar ratio lower than the calcein release assay using LUVs. This might be due to binding mostly on the outer surface of the multilamellar liposomes. This suggested that Cyt2Aa2 binds and fully covers the lipid membrane before forming protein complexes. Our data agreed with previous experiments which showed that Cyt1A aggregated into nonstoichiometric complexes in the presence of lipid bilayers [24]. A disruption of LUVs membrane integrity by the 24-kDa Cyt1A was proposed to require at least 140 molecules of membrane-bound protein [18].

### Binding and aggregation of Cyt2Aa2 on *A. albopictus* cell membrane

**Figure 5:**
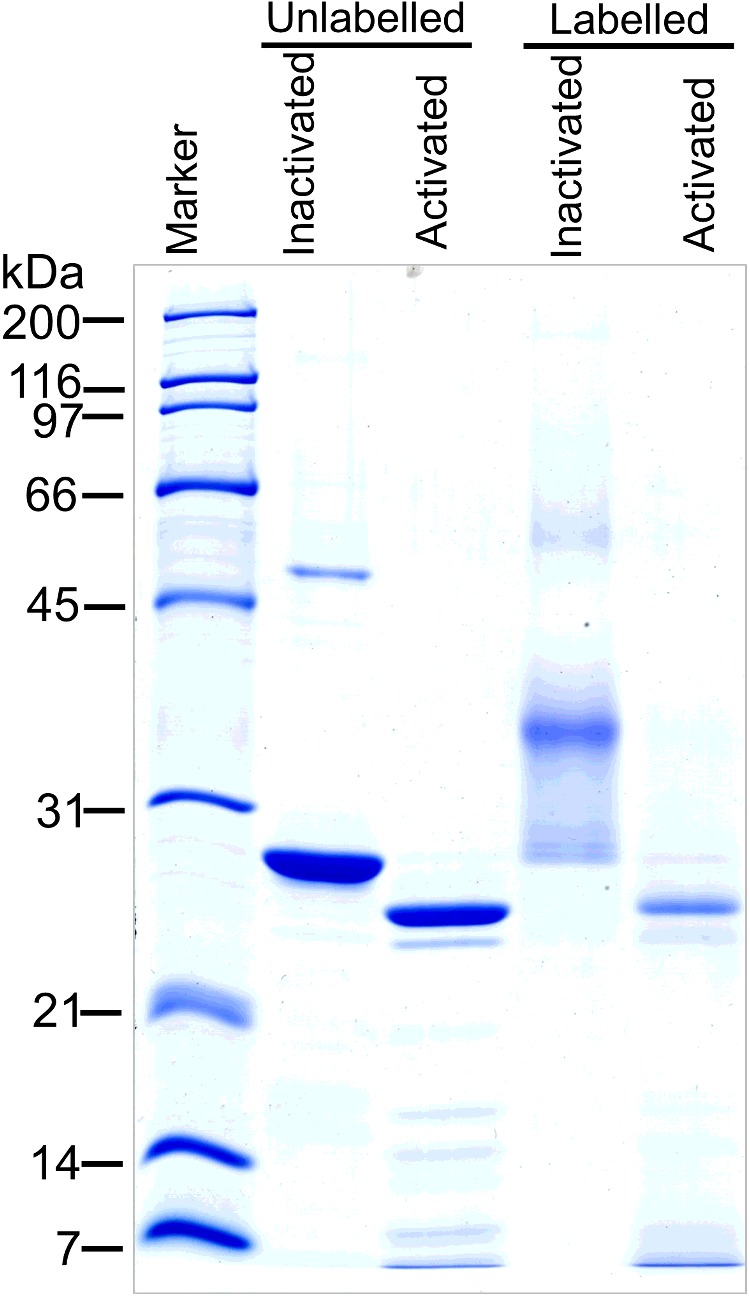
Chymotrypsin activation of labelled Cyt2Aa2 Cyt2Aa2 protoxin was activated with 2% (w/w) chymotrypsin at 37°C for 2 h. The activated protein then was analysed by 12.5% SDS/PAGE and visualized by Coomassie blue staining.

**Figure 6:**
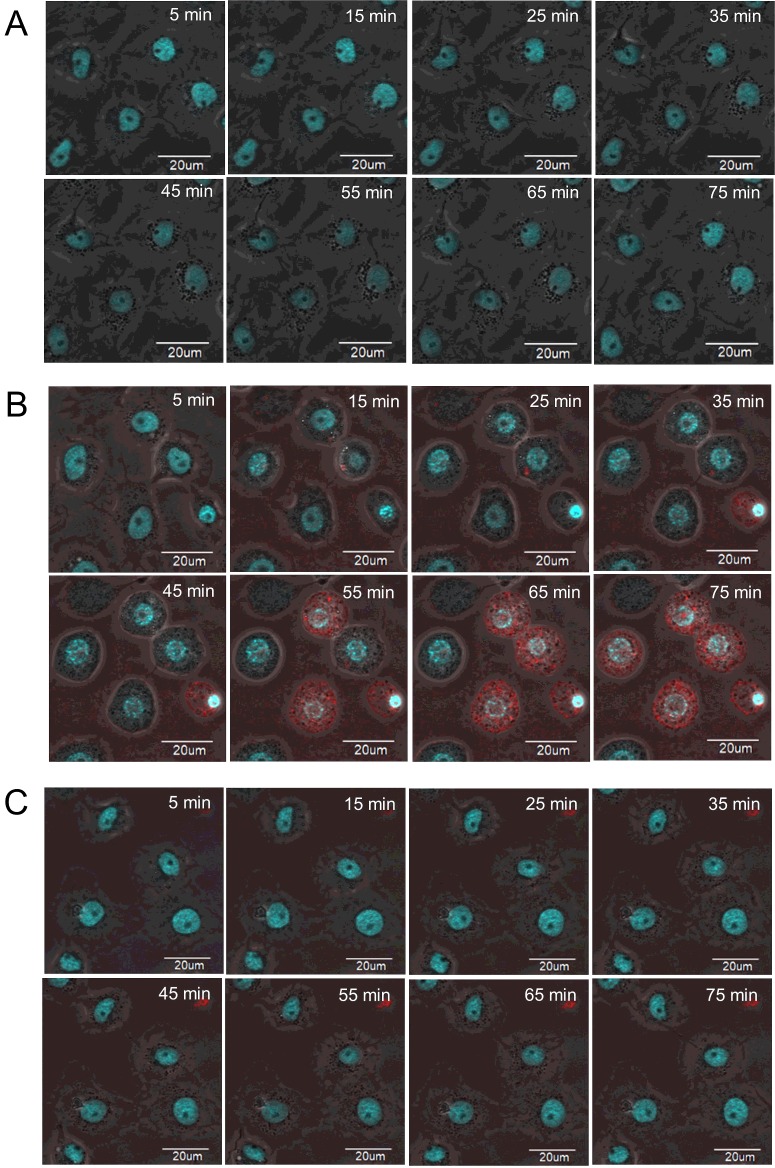
Time-course cell membrane binding of Cyt2Aa2 *A. albopictus* cells were incubated with 10 μg/ml of active labelled Cyt2Aa2 (red signal). The nucleus was stained with Hoechst (blue signal). The images were captured by a confocal microscope every 10 min at 600× magnification and displayed in a combined mode of fluorescent signal and phase contrast. (**A**) Non-treated cells, (**B**) Cyt2Aa2 wild type and (**C**) N145A inactive mutant.

**Figure 7:**
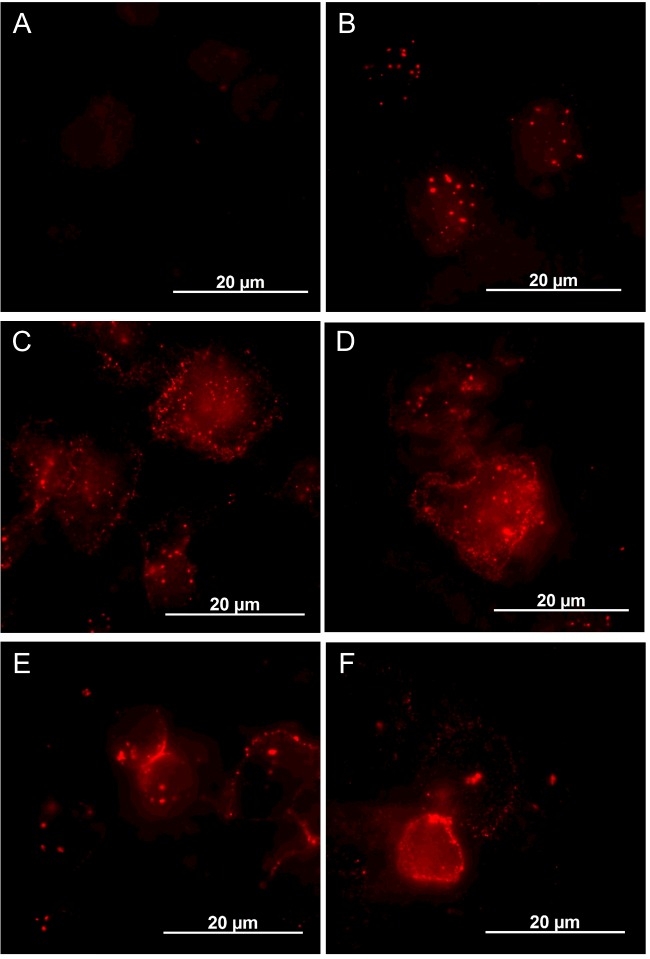
Cell membrane binding of Cyt2Aa2 detected by total internal refection fluorescent microscopy *A. albopictus* cells were incubated with 10 μg/ml of active labelled Cyt2Aa2 (red signal) at 25°C for various times. The cells were fixed with 4% paraformaldehyde and mounted with DABCO antifade. Cyt2Aa2-bound cell membrane was imaged at 1000× magnification by 640 nm exiting laser (red signal). (**A**) Non-treated cells, (**B**) 20 min of incubation of 10 μg/ml of labelled Cyt2Aa2 N145A, (**C**) 5 min of incubation of labelled Cyt2Aa2 wild type, (**D**) 10 min of incubation of labelled Cyt2Aa2 wild type, (**E**) 15 min of incubation of labelled Cyt2Aa2 wild type and (**F**) 20 min of incubation of labelled Cyt2Aa2 wild type.

The purified protoxin Cyt2Aa2 (29 kDa) was labelled by Texas red-X succinimidyl ester. The calculated labelling degree was 1.98, indicating that one molecule of Cyt2Aa2 was labelled by 1.98 molecules of dye. The labelled Cyt2Aa2 was found to show less haemolytic activity than the unlabelled sample, nevertheless the former could form the protein complexes resemble to the latter (Supplementary data 1–3). The labelled toxin had a larger molecular mass than the unlabelled toxin because of protein aggregation as shown by size exclusion chromatography (Supplementary data 4), however the labelled protein could be activated by chymotrypsin and yielded an active protein (25 kDa) similar to an unlabelled one (Figure 5). To monitor the binding of Cyt2Aa2 toxin on the cell membrane, *A. albopictus* cells were cultured and incubated with 10 μg/ml of labelled Cyt2Aa2. Fluorescence confocal microscopy images were captured every 10 min. The treated cells were observed to change their cell morphology towards a round shape concomitantly with DNA condensation at the early incubation time of 15 min. During the first 35 min of fluorescence signal monitoring Cyt2Aa2 penetrated into the cytoplasm and accumulated around the nucleus of the cell (Figure 6). The cell membrane binding of Cyt2Aa2 at early incubation times was also investigated by total internal reflection fluorescent microscopy (TIRF). The results revealed that Cyt2Aa2 bound to the cell membrane after 5, 10 and 15 min of incubation. Subsequently, the protein aggregation (large red spot on the membrane) was significantly observed at 20 min of incubation (Figure 7). Labelled mutant toxin, N145A with no lytic activity was then investigated. The TIRF experiment revealed this inactive toxin bound to the cell membrane (observed in TIRF images), but the cell morphology was not changed (observed in confocal images) as it has less ability to bind on the cell membrane than the wild-type protein [32]. These results suggested that Cyt2Aa2 bound to the cell membrane during early incubation without a significant change of cell morphology. The aggregates found at a time coincide with cell swelling which may imply protein aggregates and disruption the mosquito cell membrane. Previous studies on radio-labelled Cyt1A binding on *A. albopictus* and *Choristoneura fumiferana* cells reveals Cyt1A initially aggregated on the cell membrane when the bound toxin accumulates to a specific level [33]. Moreover, DNA condensation was observed in the Cyt1A-expressing cells of *E. coli* [34] and human leukaemic T-cells treated with the 28 kDa toxin from *Bt* subsp. *shandongiensis* [35]. The DNA condensation could be a result of either the toxicity mechanism of the toxin or cell necrosis caused by the protein treatment.

### Binding and aggregation of Cyt2Aa2 on lipid bilayers visualized by AFM

**Figure 8:**
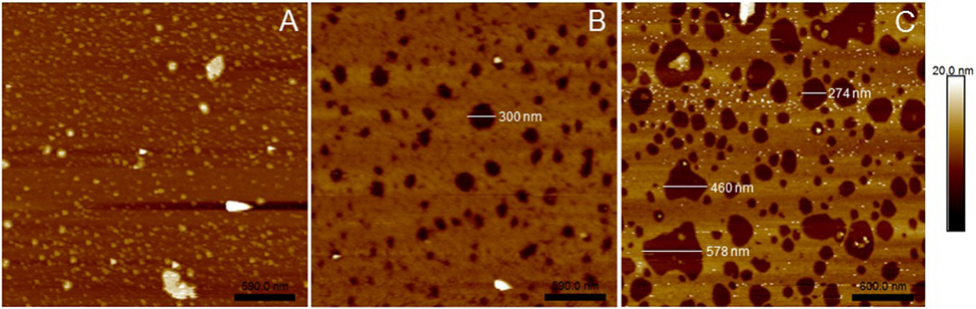
AFM topographic image of Cyt2Aa2–lipid bilayer complexes at different protein concentrations SLBs on silicon wafer were incubated with activated Cyt2Aa2 at protein concentration; 17.5 μg/ml (**A**), 25 μg/ml (**B**) and 100 μg/ml (**C**) at room temperature for 1 h. The topographic images of protein–lipid bilayer complexes were visualized by tapping mode AFM. The white bar with a number indicates the hole diameter formed on the lipid bilayer.

AFM was used to visualize the Cyt2Aa2–lipid bilayer complexes. The protein concentrations used in AFM experiment were determined based on our previous results of real time mass sensitive measurement of quartz crystal microbalance with dissipation (QCM-D). The different protein concentrations; 17.5, 25 and 100 μg/ml revealed a distinct behaviour of lipid bilayer binding. The bare silicon wafers were incubated with Cyt2Aa2 at high and low protein concentrations as a negative control which demonstrated Cyt2Aa2 did not bind on the silicon substrate (Supplementary data 5). The AFM results revealed that exposure of this lipid bilayer to Cyt2Aa2 at different protein concentrations leads to formation of distinct protein–lipid complexes (Figure 8). At 17.5 μg/ml, Cyt2Aa2 aggregated on the lipid bilayer surface as a small dot in the image. Apparently non-specific holes in the lipid bilayer were found when using toxin at higher concentrations; 25 and 100 μg/ml. At 100 μg/ml, diameters of the holes could be up to 578 nm whereas the largest hole observed when using the toxin at 25 μg/ml was 300 nm. AFM topographic images support the lipid bilayer binding of Cyt2Aa2 as suggested by haemolytic activity and calcein release assays. At low protein concentration, Cyt2Aa2 binds and aggregates on the lipid bilayer (Figure 2A). Increasing protein concentrations of Cyt2Aa2 leads to membrane disruption of the lipid bilayer which is shown by the observed calcein leakage at suitable protein/lipid ratio. Moreover, the area of membrane disruption increased with protein concentration. Accordingly, PEG4000 could not completely inhibit the haemolytic activity at high protein concentrations as Cyt2Aa2 can form holes with diameters up to ∼600 nm which are large enough for either haemoglobin (*R*_h_ ≈ 3.4 nm) or PEG4000 (*R*_h_ ≈ 1.8 nm) to pass through the lipid membrane. (The radius of molecules are calculated following Einstein viscosity relation.) The membrane leakages formed at high protein concentrations can allow not only soluble proteins inside the cell to leak out but also some cell organelles e.g. ribosome (diameter ∼20–30 nm) and small lysosomes (diameter ∼50–3000 nm) to move out via these leakages. These AFM images suggest that Cyt2Aa2 binds and aggregates on the lipid bilayer at low protein concentrations whereas disruption of the lipid membrane by Cyt2Aa2 took place when the bound toxin accumulates to a certain level at higher protein concentrations. This result corresponds with QCM-D data which the dissipation values infer to a distinct behaviour of lipid bilayer binding at different protein concentrations (10 and 100 μg/ml). On the other side, the deposited mass, lipid bilayer and Cyt2Aa2 did not lose from the surface when applying the toxin at 100 μg/ml implying the mechanism of Cyt2Aa2 differs from a detergent-like model [36].

## CONCLUSIONS

The mechanism of haemolytic activity of Cyt2Aa2 toxin was investigated. We demonstrated that its membrane disruption mechanism is different from the action of general pore-forming proteins. Our study showed that osmotic stabilizing agent, PEG4000 partially inhibits haemolysis. The lipid membrane binding and protein complex formation analysed on the lipid vesicles revealed the monomeric binding of toxin under specific protein/lipid molar ratios. Subsequent aggregation and formation of non-specific large protein complexes at a critical protein/lipid molar ratio then disrupted the lipid membrane integrity as the entrapped molecules are escaped from the vesicles. Our membrane binding study on *A. albopictus* cell culture revealed the binding of Cyt2Aa2 at an early step of incubation. Cell swelling was then induced, concomitant with Cyt2Aa2 aggregation on the lipid membrane. Finally, protein binding on lipid membrane and protein aggregation was determined by AFM. The topographic surfaces suggest that at low protein concentration Cyt2Aa2 binds on the lipid membrane whereas non-specific hole formation took place when using higher protein concentrations. These results suggest that the lipid membrane disruption of Cyt2Aa2 is a protein concentration dependent phenomenon. The membrane lesion formed at the high protein concentration implies that Cyt2Aa2 binds and aggregates on the membrane and subsequently form a non-specific hole when the bound toxin accumulated to a certain level. The hole is enlarged upon increasing the protein concentration. This phenomenon may resemble that of streptolysin O in which the enlarged pore is formed by addition of toxin monomers into the pore complex [37,38].

## Abbreviations

*Bt*: *Bacillus thuringiensis*
Cry: crystal toxin
Cyt: cytolytic toxin
Cyt2Aa2: cytolytic toxin from *Bacillus thuringiensis* subsp. *darmstadiensis*
Cyt1Aa and Cyt2Ba: cytolytic toxin from *Bacillus thuringiensis* subsp. *israelensis*
DABCO: 1,4-diazobizyclo(2,2,2) octane
LUV: large unilamellar vesicle
SLB: supported lipid bilayer
TIRF: total internal reflection fluorescent
